# Characterization of trehalolipid biosurfactant produced by the novel marine strain *Rhodococcus* sp. SP1d and its potential for environmental applications

**DOI:** 10.1186/s12934-023-02128-9

**Published:** 2023-07-13

**Authors:** Marco Andreolli, Valeria Villanova, Serena Zanzoni, Mariapina D’Onofrio, Giovanni Vallini, Nicola Secchi, Silvia Lampis

**Affiliations:** 1 VUCC-DBT Verona University Culture Collection, Department of Biotechnology, University of Verona, Strada le Grazie, 15, Verona, 37134 Italy; 2 Department of Biotechnology, University of Verona, Strada le Grazie, 15, Verona, 37134 Italy; 3 Department of Biological, Chemical and Pharmaceutical Sciences and Technologies (STEBICEF), University of Palermo, Palermo, Italy; 4 Centro Piattaforme Tecnologiche, University of Verona, Verona, Italy; 5 Eurovix S.p.A, Viale Mattei 17, Entratico, Bergamo, 24060 Italy

**Keywords:** *Rhodococcus* sp., Biosurfactants, Trehalolipids, Emulsification, Nuclear magnetic resonance spectroscopy, Biofilm, Bioremediation

## Abstract

**Background:**

Biosurfactants are surface-active compounds with environmental and industrial applications. These molecules show higher biocompatibility, stability and efficiency compared to synthetic surfactants. On the other hand, biosurfactants are not cost-competitive to their chemical counterparts. Cost effective technology such as the use of low-cost substrates is a promising approach aimed at reducing the production cost. This study aimed to evaluate the biosurfactant production and activity by the novel strain *Rhodococcus* sp. SP1d by using different growth substrates. Therefore, to exploit the biosurfactant synthesized by SP1d for environmental applications, the effect of this compound on the bacteria biofilm formation was evaluated. Eventually, for a possible bioremediation application, the biosurfactant properties and its chemical characteristics were investigated using diesel as source of carbon.

**Results:**

*Rhodococcus* sp. SP1d evidence the highest similarity to *Rhodococcus globerulus* DSM 43954^T^ and the ability to biosynthesize surfactants using a wide range of substrates such as exhausted vegetable oil, mineral oil, butter, n-hexadecane, and diesel. The maximum production of crude biosurfactant after 10 days of incubation was reached on n-hexadecane and diesel with a final yield of 2.38 ± 0.51 and 1.86 ± 0.31 g L^− 1^ respectively. Biosurfactants produced by SP1d enhanced the biofilm production of *P. protegens* MP12. Moreover, the results showed the ability of SP1d to produce biosurfactants on diesel even when grown at 10 and 18 °C. The biosurfactant activity was maintained over a wide range of NaCl concentration, pH, and temperature. A concentration of 1000 mg L^− 1^ of the crude biosurfactant showed an emulsification activity of 55% towards both xylene and olive oil and a reduction of 25.0 mN m^− 1^ of surface tension of water. Eventually, nuclear magnetic resonance spectroscopy indicated that the biosurfactant is formed by trehalolipids.

**Conclusions:**

The use of low-cost substrates such as exhausted oils and waste butter reduce both the costs of biosurfactant synthesis and the environmental pollution due to the inappropriate disposal of these residues. High production yields, stability and emulsification properties using diesel and n-hexadecane as substrates, make the biosurfactant produced by SP1d a sustainable biocompound for bioremediation purpose. Eventually, the purified biosurfactant improved the biofilm formation of the fungal antagonistic strain *P. protegens* MP12, and thus seem to be exploitable to increase the adherence and colonization of plant surfaces by this antagonistic strain and possibly enhance antifungal activity.

## Introduction

Surface-active surfactants consist of hydrophobic and hydrophilic moieties, exhibiting a wide range of chemical structures, such as lipopeptides, glycolipids, polysaccharide-proteins, and nucleolipids [1, 2]. These compounds have several industrial-relevant properties such as reduction of the viscosity of crude oil, emulsification, foaming, wetting, phase separation, and cleansing [3, 4]. Moreover, both synthetic and biological surfactants have an impact on biofilm growth in a wide range of medical, and environmental bacterial strains [5]. Compared to synthetic surfactants, microbiologically synthesized biosurfactants show many advantages, including structural diversity, low toxicity, good biodegradability, stability at extreme temperatures, salinity, and pH values [6]. Biosurfactants are becoming more popular as alternatives to synthetic surfactants in a wide range of applications, such as pharmaceuticals, cosmetics, oil, detergents, paint, textiles, food processing, agriculture, and pollution removal [3, 7, 8]. The most notable bacterial genera able to produce surface-active compounds are *Pseudomonas* sp. (rhamnolipids, ornithine lipids, carbohydrate lipids), *Bacillus* sp. (surfactins, lipopeptides), *Acinetobacter* sp. (glycolipids, lipopeptides, rhamnolipids, and glycolipoproteins) and *Rhodococcus* sp. (trehalolipids) [2, 4]. Several species belonging to *Rhodococcus* genus were identified as biosurfactant producers such as *R. erythropolis*, *R. ruber, R. fascians*, *R. longus*, *R. wratislaviensis, R. equi, R. rhodochrous* and *R. opacus* [9–11]. Trehalolipid biosurfactants produced mainly by this genus are widely applied as emulsifying compounds with application in oil recovery from reservoirs and in bioremediation protocols of oil-contaminated soils [12]. Moreover, trehalolipids can be potentially used in medical fields as antiviral, antimicrobial, anticancer, or immunomodulating factor [13, 14]. Trehalolipids are mainly produced by organisms utilizing hydrophobic substrates such as n-alkane, crude and vegetable oils [6, 13], however, their production has also been observed employing soluble substrates [15–17]. Marine environments are rich in microorganisms that have evolved unique physiological and metabolic characteristics [18]. Several studies showed this habitat as a promising source for the discovery of novel bacterial strains able to produce biosurfactants [19–22].

This study aimed to evaluate the properties of biosurfactants produced by the novel marine strain *Rhodococcus* sp. SP1d by using different carbon sources such as low-cost substrates to possibly reduce the production costs. Moreover, their potential environmental applications (in bioremediation protocols and as biocontrol agents) were evaluated. The investigation includes: *(i)* a preliminary taxonomic identification of SP1d strain and *(ii)* the characterization of biosurfactants produced by using different carbon substrates. Moreover, in order to potentially exploit the biosurfactant synthesized by SP1d for environmental applications *(iii)* the effect of *Rhodococcus* sp. SP1d biosurfactant on the biofilm production of a plant growth promoting (PGP) and fungal antagonistic bacterium such as *Pseudomonas protegens* MP12 [23] was evaluated. Finally, *(iv)* for a potential bioremediation application, the biosurfactant properties and its chemical characteristics were investigated using diesel as sole source of carbon and energy.

## Results

### Identification of Rhodococcus sp. SP1d

**Fig. 1:**
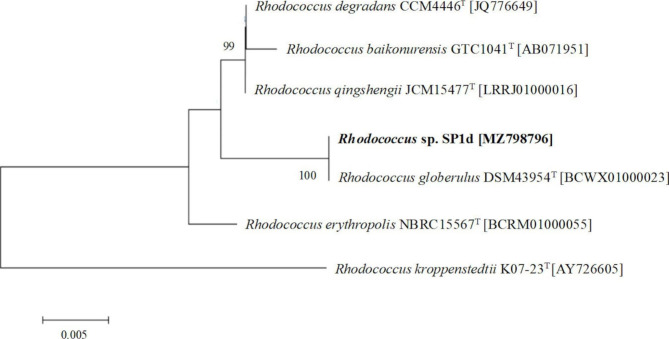
Phylogenetic tree generated by 16 S rRNA sequence retrieved from *Rhodococcus* sp. SP1d. Outgroup = *Rhodococcus kroppenstedtii* K07-23T. Bootstrap values are shown at branch nodes and are based on 1000 replicates. The scale bar indicates 0.005 substitutions per nucleotide position

The sequence of the 16 S rRNA gene (1359 bp) of SP1d strain was determined and the phylogenetic tree was constructed (Fig. 1). The results revealed an identity of 100% with *Rhodococcus globerulus* DSM 43954^T^. Moreover, significant identity was found with *Rhodococcus qingshengii* JCM 15477^T^ (99.48%), *Rhodococcus degradans* CCM 4446^T^ (99.04%), *Rhodococcus baikonurensis* GTC 1041^T^ (99.04%), *Rhodococcus baikonurensis* GTC 1041^T^ (98.77%) and *Rhodococcus erythropolis* NBRC 15567^T^ (98.38%).

### Growth tests in presence of different hydrophobic substrates

**Fig. 2:**
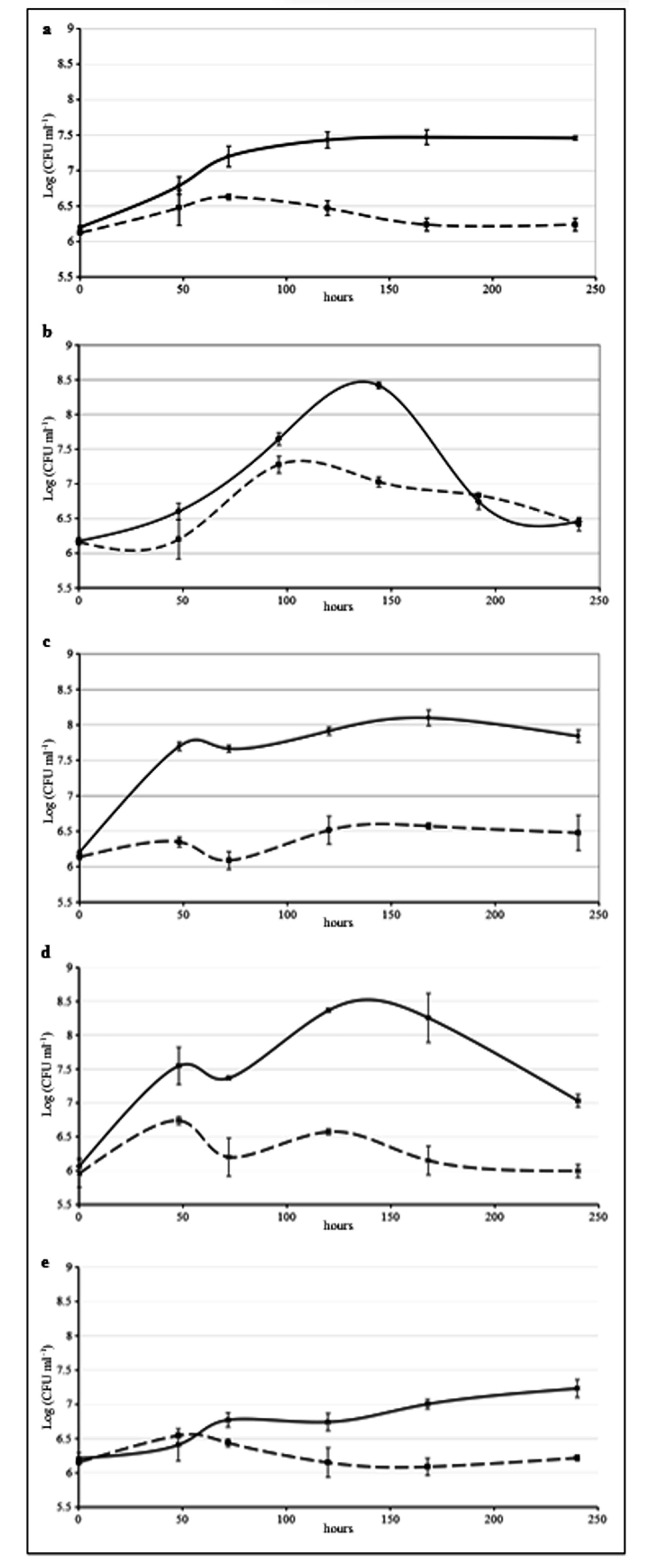
Growth curves of *Rhodococcus* sp. SP1d *Continuous lines*: bacterial growth in presence of 1% of (**a**) n-hexadecane, (**b**) diesel, (**c**) butter, (**d**) exhausted vegetable oil, and (**e**) motor oil. *Dotted lines*: bacterial growth in absence of substrate. Each curve is based on the results of three replicates

Results showed that *Rhodococcus* sp. SP1d was able to grow on n-hexadecane, diesel, butter, exhausted vegetable oil, and motor oil as the sole sources of energy and carbon, although the growth on this latter substrate proceeds with a lower yield (Fig. 2). In presence of diesel and exhausted oil the strain reached the maximum growth after about 120 h with a cell concentration of 8.5 log_10_ (CFUs ml^− 1^). Growth curves in DM supplied with butter showed an exponential phase for SP1d that lasted about 48 h and reached the maximum biomass production -- ~8 log_10_ (CFUs ml^− 1^) -- after 170 h. Eventually, the strain is also capable of utilizing motor oil but with the cell concentration increasing only by about 1.5 and 1 order of magnitude respectively over 10 days of incubation.

### Evaluation of biosurfactant production by SP1d strain grown on different substrates

Production of biosurfactant and its surface tension reduction, oil-displacement, emulsification ability, and the measurement of turbidity of the resulting emulsion were evaluated for *Rhodococcus* sp. SP1d grown using n-hexadecane, diesel, butter, exhausted vegetable oil, and motor oil as substrates after 10 days of incubaton (Table 1). The maximum production of crude biosurfactant after 10 days was reached on n-hexadecane and diesel with a final yield of 2.38 ± 0.51 and 1.86 ± 0.31 g L^− 1^ respectively. Moreover, biosurfactant was synthetized using exhausted vegetable oil (1.61 ± 0.23 g L^− 1^), butter (0.81 ± 0.29 g L^− 1^) and mineral oil (0.31 ± 0.25 g L^− 1^) as source of carbon and energy. Thus, a reduction in surface tension in cell-free medium was observed when SP1d strain was grown in n-hexadecane (14 mN m^− 1^), diesel (15.8 mN m^− 1^) and exhausted vegetable oil (3.96 mN m^− 1^). On the other hand, surface tension measurements were altered by the viscous nature of butter and mineral oil and the bioemulsified cultures [17].

The oil-displacement test showed a large oil-free clearing zone of 13.6 ± 1.53 and 9.0 ± 1.41 mm by using n-hexadecane and diesel as carbon and energy sources respectively, while biosurfactant produced by SP1d strain grown on exhausted oil displaced circle with 4.5 ± 0.71 mm of diameter. Employing butter and motor oil as substrates the diameter of the clear zone decreased to 3.2 ± 1.05 and 2.5 ± 0.92 mm respectively. Similarly, the highest % emulsification activity (EA24) was observed for the biosurfactant synthesized by *Rhodococcus* sp. SP1d grown on n-hexadecane with values ranging from 56.15 to 62.30 followed by that produced in presence of diesel with a percentage in the range of 51.54–44.61%, among the compounds tested. The turbidity of the emulsion obtained for the biosurfactant produced using n-hexadecane as growth substrate was 53.50 ± 4.24, 24.52 ± 4.95, 23.53 ± 3.26, and 14.00 ± 2.83 with xylene, diesel, hexane, and olive oil respectively. On the other hand, the turbidity observed using diesel as carbon source was with xylene (20.7 ± 2.54) followed by olive oil (15.25 ± 2.47), diesel (12.95 ± 1.76) and hexane (5.30 ± 0.14). Therefore, the presence of exhausted oil as substrates leads to a % EA24 between 40.769 and 37.692%. The turbidites of the emulsions were found to be OD_600_ 9.75 ± 3.18, 5.375 ± 0.67, 5.1 ± 0.84, and 2.75 ± 0.49 by using xylene, diesel, olive oil, and hexane respectively. The % EA24 recorded by *Rhodococcus* sp. SP1d grown on butter were in the range of 40.769–22.308% with a turbidity of OD_600_ 5.95 ± 0.35 with diesel and 3.80 ± 0.42 with hexane. The volume of the emulsions formed with olive oil and xylene was too low to measure the turbidity. Eventually, the emulsification activity dropped when SP1d was grown on motor oil with a % EA24 between 3.846 ± 1.08 and 6.923 ± 2.17, with no possibility to determine its turbidity. All the emulsions were stable for more than 15 days.

**Table 1:** Biosurfactant production (g L^− 1^), its surface tension reduction (mN m^− 1^), oil displacement test (mm), emulsification activity (% EA24) and determination of the turbidity of the emulsion (OD_600_) for *Rhodococcus* sp. SP1d grown using different hydrophobic substrates after 10 days of incubation (n.d.) not determined. (--) Surface tension measurements were confounded by the viscousnature of butter and mineral oil and the bioemulsified cultures

	Growth substrates				
	n-hexadecane	Diesel	Mineral oil	Exhausted vegetable oil	Butter
**Purified biosurfactant (g L** ^**-1**^ **)**	2.38 ± 0.51	1.86 ± 0.31	0.31 ± 0.25	1.61 ± 0.23	0.81 ± 0.29
**Surface tension (mN m** ^**-1**^ **)** Reference culture Bioemulsified culture Difference	60.9 ± 0.30 46.9 ± 1.34 14	58.6 ± 1.17 42.8 ± 2.20 15.8	34.89 ± 1.92 32.75 ± 0.44 --	53.32 ± 0.17 49.36 ± 0.33 3.96	43.35 ± 0.21 44.17 ± 0.34 --
**Oil displacement test (mm)**	13.6 ± 1.53	9.0 ± 1.41	2.5 ± 0.92	4.5 ± 0.71	3.2 ± 1.05
**Emulsification activity (% EA24)**					
Diesel	56.15 ± 3.26	46.92 ± 3.26	3.84 ± 1.08	40.00 ± 2.17	40.76 ± 7.61
Olive oil	61.53 ± 4.35	44.61 ± 4.35	6.92 ± 2.17	40.76 ± 3.26	22.30 ± 5.43
Hexane	62.30 ± 2.17	46.92 ± 7.61	5.38 ± 1.08	39.23 ± 3.26	40.00 ± 2.17
Xylene	57.69 ± 1.26	51.53 ± 3.23	4.61 ± 4.35	37.69 ± 5.43	24.61 ± 2.17
**Turbidity of the emulsion (OD**_**600**_ **)**					
Diesel	24.52 ± 4.95	12.95 ± 1.76	n.d.	5.37 ± 0.67	5.95 ± 0.35
Olive oil	14.00 ± 2.83	15.25 ± 2.47	n.d.	5.10 ± 0.84	n.d.
Hexane	23.53 ± 3.26	5.30 ± 0.14	n.d.	2.75 ± 0.49	3.80 ± 0.42
Xylene	53.50 ± 4.24	20.7 ± 2.54	n.d.	9.75 ± 3.18	n.d.

### Monitoring of the biosurfactant production during Rhodococcus sp. SP1d growth

**Fig. 3:**
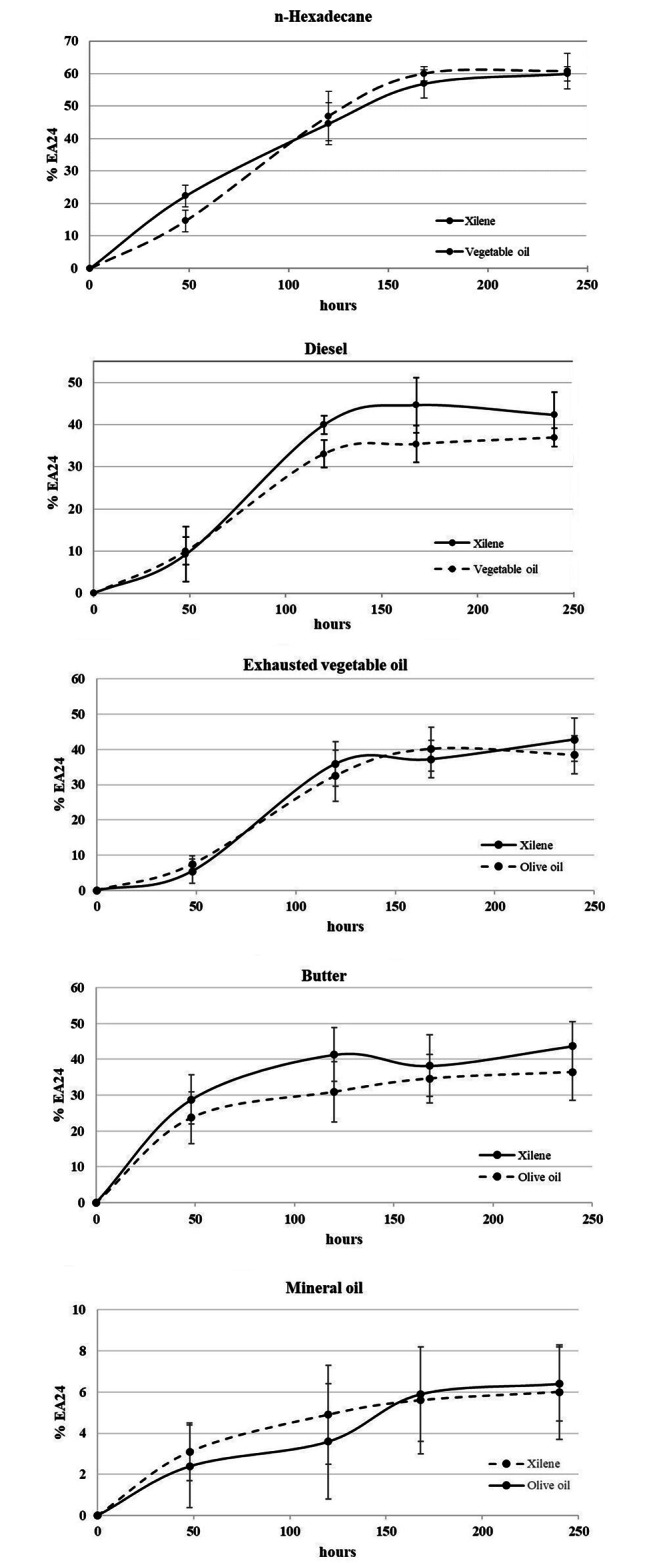
Emulsification activity (% EA24) values measured on cell-free media of *Rhodococcus* sp. SP1d grown on n-hexadecane, diesel, mineral oil, exhausted vegetagle oil and butter as the sole source of carbon and energy after 0, 48, 120, 168, and 240 h of incubation. Error bars represent the values of three independent experiments

The biosurfactant production was monitored during the growth of *Rhodococcus* sp. SP1d cultivated on n-hexadecane, diesel, mineral oil, exhausted vegetable oil and butter as the sole sources of carbon and energy. The % EA24 was calculate on cell-free supernatant after 0, 48, 120, 168, and 240 h (Fig. 3). The results evidenced an exponential trend of the % EA24 until about 168 h of incubation, followed by a stationary phase until the end of the experimentation.

*Effect of biosurfactants on biofilm production of Pseudomonas protegens* MP12.

**Fig. 4:**
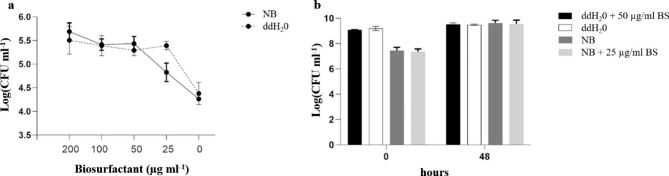
(**a**) Effect of different concentrations of biosurfactants (BS) from *Rhodococcus* sp. SP1d on the biofilm production of growing (NB) and resting cells (ddH_2_O) of *P. protegens* MP12 after 48 h of incubation at 27 °C. (**b**) Growth of planktonic cells after 48 h of incubation in growing (NB) and resting (ddH_2_O) ccondition in presence and absence of biosurfactant (BS)

Biosurfactant produced by *Rhodococcus* sp. SP1d was tested for their potential on improving adherence to plant surfaces (i.e. enhancing the biofilm production) in the *Pseudomonas protegens* MP12 [23]. Our results showed that *Pseudomonas protegens* MP12 produced about log_10_ 4.5 CFU mL^− 1^ of biofilm in both growing (i.e., Nutrient broth) and resting (i.e. ddH_2_O) cells in control condition after 48 h at 27 °C. The addition of 25 and 50 µg mL^− 1^ of biosurfactant enhanced the biofilm production for growing and resting cells respectively. Higher concentrations (up to 200 µg mL^− 1^) of the biosurfactant did not increase the biofilm production (Fig. 4a). By contrast the biosurfactant addition did not affect the growth of planktonic cells either in growing or resting cells (Fig. 4b). Our results showed the potential use of the biosurfactants produced by *Rhodococcus* sp. SP1d to increase the biofilm production in the PGP bacterium *P. protegens* MP12.

### Investigation on the biosurfactant production and activity using diesel as substrate

#### Effects of the temperature on SP1d growth and biosurfactant production

Although no bacterial growth was observed at both at 18 and 10 °C (data not shown), the results evidenced a % EA24 in hexane of 5.71 ± 4.04 and 44.29 ± 2.02 and an oil-displacement of 1.75 ± 2.06 and 4.75 ± 1.26 mm at 10 and 18 °C respectively.

### Effects of sodium chloride, pH. and temperature on biosurfactant stability

**Fig. 5:**
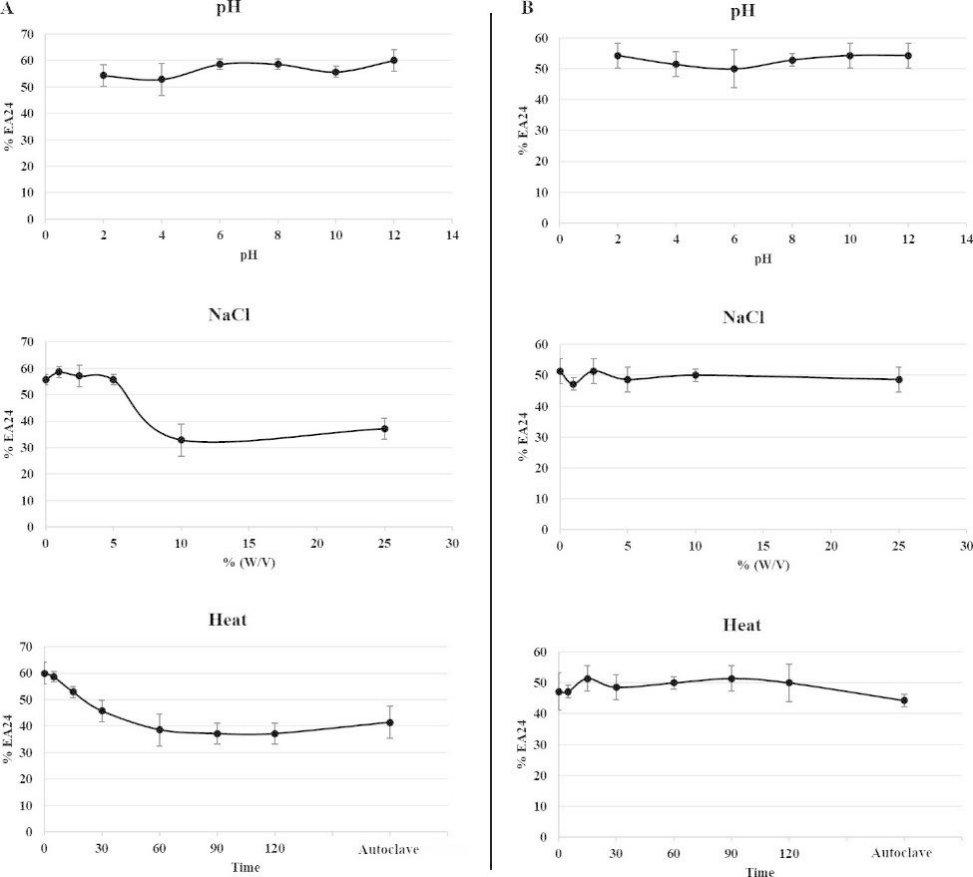
Effect of pH, NaCl, and heat on the stability of (**A**) extracted crude biosurfactant and (**B**) cell-free medium measured by % EA24 tested against xylene. Error bars represent the values of three independent experiments

The effects of NaCl, pH, and heating on biosurfactant stability measured as % EA24 were shown in Fig. 5. The biosurfactant activity derived from cell-free medium was maintained over a wide range of NaCl concentrations, pH values and heating times with minimal deviation in the % EA24. On the other hand, the extracted crude biosurfactant showed no significant differences from 2 to 12 pH values, but % EA24 values dropped in presence of 10% or more of NaCl. Eventually, the emulsification activity decreased from 60 to about 40% after 60 min of heating.

### Surface tension and emulsification abilities of extracted crude biosurfactant

A reductions of 25.0 mN m^− 1^ of surface tension was observed in water supplied with 1000 mg L^− 1^ of purified biosurfactant compared to bidistilled water: from 72.84 ± 0.11 mN m^− 1^ to 47.82 ± 2.24 mN m^− 1^.

**Fig. 6:**
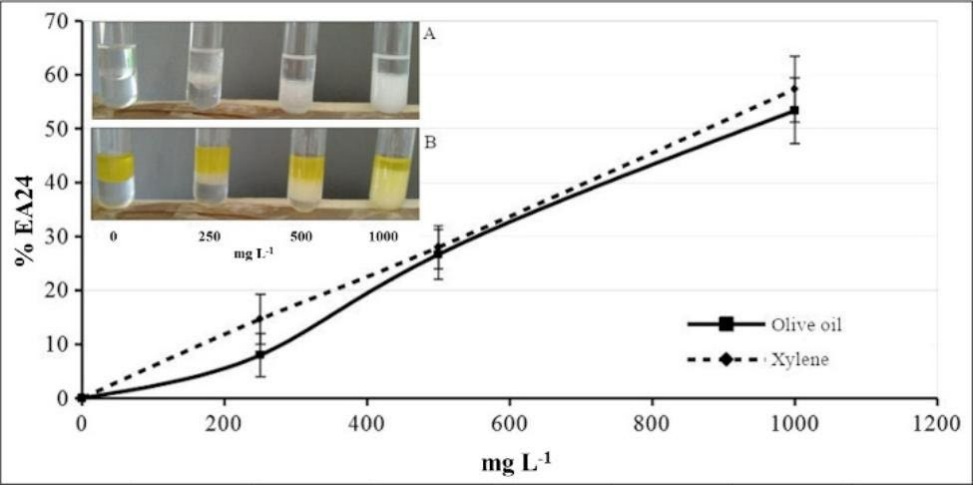
Emulsification activity (% EA24) tested again olive oil and xylene of biosurfactant purified from *Rhodococcus* sp. SP1d strain grown on diesel

The % EA24 activity of extracted crude biosurfactant was tested (Fig. 6). The results evidenced a linear curve between the concentration of crude biosurfactant and % EA24 values reaching a percentage of emulsification activity of about 55% either with xylene or olive oil using 1000 mg L^− 1^ of crude biosurfactant.

### Chemical characterization of biosurfactants produced by SP1d strain

The biosurfactants obtained growing *Rhodococcus* sp. SP1d on diesel as the sole source of carbon were analyzed using Nuclear Magnetic Resonance (NMR) spectroscopy. High-resolution NMR spectroscopy is an exquisite technique to analyze molecules composition even in complex mixtures. NMR-active nuclei are extremely sensitive to their chemical environment, therefore the analysis of NMR spectra allows structural elucidation, and qualitative information about the molecules under investigation.

One dimensional ^1^ H NMR spectrum acquired on the crude extracts of biosurfactants dissolved in D_2_O, shows a variety of signals with chemical shifts in the 5.5–3.3 and 2-0.8 ppm regions characteristic of the presence of long hydrocarbon chains and carbohydrate rings. However, the ^1^ H NMR spectrum has limited chemical shift dispersion and therefore is difficult to interpret. Wetherefore recorded 2D homo and heteronuclear spectra with the aim to further characterize the chemical nature of the biosurfactants.

**Fig. 7:**
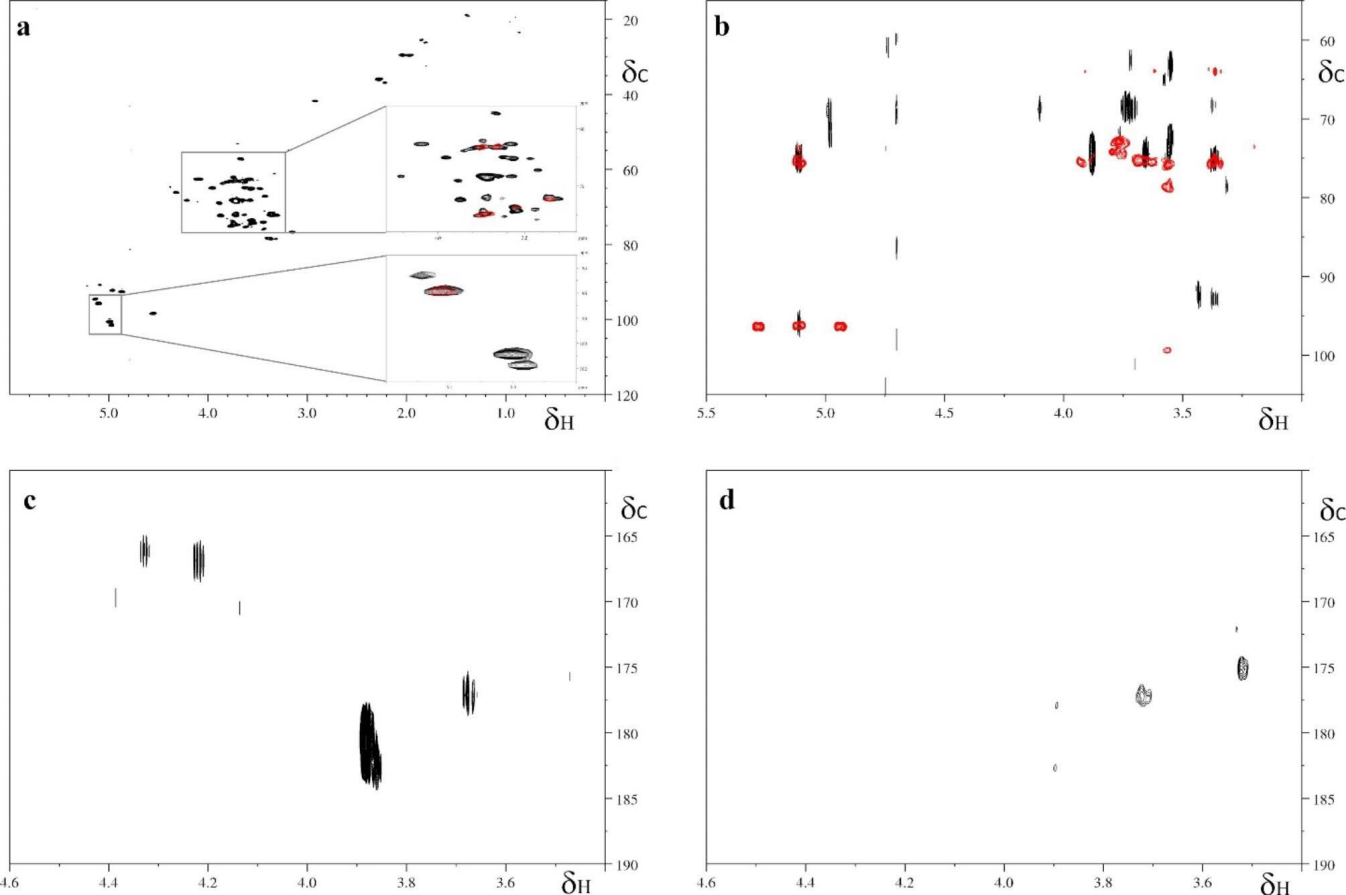
Heteronuclear NMR spectra acquired on biosurfactants dissolved in D_2_O. (**a**) ^1^ H-^13^ C HSQC spectrum of the crude extracts of biosurfactants. The insets show a zoom of the spectrum of the biosurfactants (black) superimposed with the ^1^ H-^13^ C HSQC spectrum of trehalose deposited in the BMRB (red). (**b**) Superimposition of a region of the ^1^ H-^13^ C HMBC spectra of biosurfactants (black) and of trehalose deposited in the BMRB. (**c**, **d**) Zoom of the carbonyl region of the ^1^ H-^13^ C HMBC spectra of crude biosurfactants (**c**), and after basic hydrolysis (**d**). All the spectra were recorded at 25 °C

The ^1^ H-^13^ C HSQC spectrum of the crude extracts of biosurfactants dissolved in D_2_O, showed several dispersed and resolved NMR signals in the range of 6.5–0.5 ppm in the ^1^ H dimension and 112–118 ppm in the ^13^ C dimension. The closer inspection of the signals in the ^1^ H shift range of 5.5 to 3 ppm and the comparison with the deposited spectra of standard solutions of pure substance in the Biological Magnetic Resonance Data Bank [24], allowed the identification of the main carbohydrate as the disaccharide trehalose (Fig. 7a). The analysis of the signals allowed to clearly identify the characteristic resonances of the anomeric carbon and proton (δ_C1_ 96 ppm, δ_H1_ 5.106 ppm) and the peaks of the methylene carbons C6 (δ_C6_ 63.34 ppm, δ_H6_ 3.77 ppm and δ_C6_ 63.12 ppm, δ_H6_ 3.66 ppm) [25]. The presence of the trehalose moiety is further confirmed by the analysis of the ^1^ H-^13^ C HMBC spectrum, which provides long-range correlations typical of the trehalose carbohydrate (Fig. 7b). The resonances observed in the anomeric region between 95 and 105 ppm of the ^1^ H-^13^ C HSQC spectrum (Fig. 7a inset) suggested the presence of additional carbohydrate moieties (four different pyranoside rings) however it was not possible to clearly identify signal patterns. The presence of the trehalose moiety is confirmed also by homonuclear 2D ^1^ H-^1^ H TOCSY spectrum (data not shown).

Interestingly, long-range correlations in the ^1^ H-^13^ C HMBC spectrum showed two signals at 165.74 ppm and two at 180.94 and 184.44 ppm, indicative of the presence of at least four ester groups (Fig. 7c). It is worth noticing that the signals at ~ 165 ppm (^13^ C dimension) are absent in the ^1^ H-^13^ C HMBC spectrum recorded on the aqueous phase obtained after biosurfactant basic hydrolysis (Fig. 7d); this observation suggests that esterification of hydroxyl groups of the trehalose rings and the carbonyl carbons of the fatty acids had occurred. However, due to the crowded spectrum, we could not assign the exact ^3^ J H,C correlations between the carbohydrate and the hydrocarbon chain.

The spectra of the crude biosurfactants dissolved in D_2_O revealed also characteristic ^1^ H NMR signals with chemical shifts at ~ 0.8 ppm for the methyl group (–CH_3_), ~ 1.2 ppm for the long hydrocarbon chains (–CH_2_)_*n*_–, and ~ 2.5 ppm for (–CH_2_–COO–), however, the 2D spectra recorded on the same sample were not informative.

**Fig. 8:**
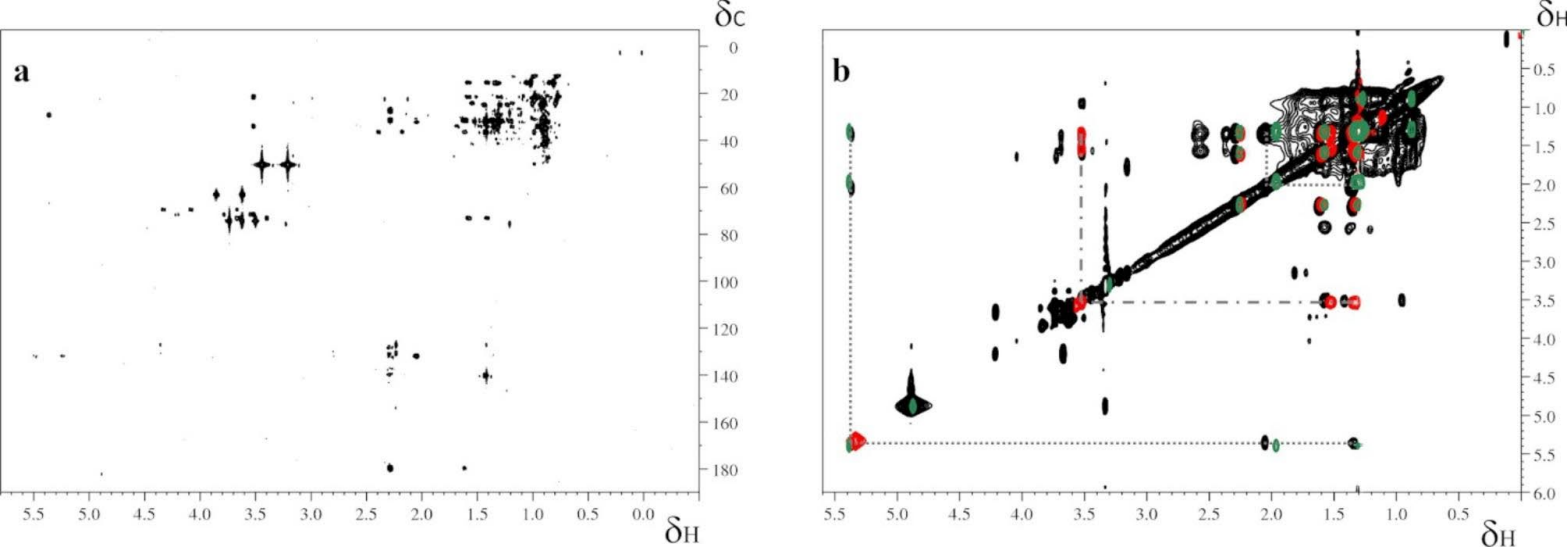
(**a**) ^1^ H-^1^ H TOCSY spectrum acquired on the organic phase obtained after basic hydrolysis of the biosurfactants. (**b**) Superimposition of the zoom of the ^1^ H-^1^ H TOCSY spectrum of biosurfactants (black) with ^1^ H-^1^ H TOCSY spectra of oleic acid (green) and 16-hydroxypalmitic acid (red) deposited in the BMRB. Dashed lines indicate typical cross peaks patterns of vinylic, allylic, and hydrocarbon chains (…) and of hydroxylated fatty acids (_._). All the spectra were recorded at 25 °C

To identify the fatty acid moieties further NMR spectra were acquired on the organic phase obtained after basic hydrolysis of the biosurfactants, dissolved in methanol. The ^1^ H-^13^ C HSQC spectrum shows the expected signals for long hydrocarbon chains, and a signal at 5.36 and 132.07 in the ^1^ H and ^13^ C carbon dimensions, respectively (Fig. 8a) corresponding to a vinylic hydrogen in the fatty acid chain. The presence of the unsaturation is also confirmed by analyzing the ^1^ H-^1^ H TOCSY spectrum where a characteristic pattern of cross-peaks of vinylic (δ_H_ 5.39 ppm), allylic (δ_H_ 2.05 ppm), and hydrocarbon chains (δ_H_ 1.34 ppm) protons is easily identified (Fig. 8b) [26]. By a closer inspection of the ^1^ H-^1^ H TOCSY spectrum, it was also possible to identify the peak at 3.52 ppm characteristic of hydroxylated fatty acids with a crosspeak at δ_H_ 3.52/0.96 ppm indicative of the hydroxylation close to the methyl group of the fatty acid (Fig. 8b). Overall NMR analysis allowed to conclude that the biosurfactant produced growing *Rhodococcus* sp. SP1d on diesel is formed by a trehalose moiety and long-chain fatty acids saturated, unsaturated and hydroxylated.

## Discussion

The marine environment represents a huge source of bacteria that can be potentially exploited for the synthesis of novel natural molecules that can be utilized for commercial aims. However, biosurfactants produced by strains isolated from marine habitats are poorly investigated in comparison to terrestrial strains [27]. Microorganisms that are exposed to hydrocarbon-polluted sites produce surface-active molecules to increase their bioavailability [28]. To date, marine strains able to produce biosurfactants belong to different genera including *Marinomonas* sp., *Bacillus* sp., *Acinetobacter* sp, *Cobetia* sp, *Catenovulum* sp., *Serratia* sp., *Pseudomonas* sp., *Pseudoalteromonas* sp., *Glaciecola* sp., *Alteromonas*, *Halomonas* and *Alcanivorax* [29–32]. Moreover, *Rhodococcus* strains able to produce biosurfactants were already isolated from marine environments: Dang et al. [29] found that, among the isolated strains, two strains belonging to this genus were the best at reducing the surface tension of the culture medium and White et al. [22] isolated *Rhodococcus* sp. PML026 with high production of biosurfactants. Indeed, several species belonging to *Rhodococcus* genus – isolated both from terrestrial and marine environments – were identified as biosurfactant producers [9–11] but, at the best of the author’s knowledge, this is the first manuscript that described the production and its potential agro-industrial applications of biosurfactants from a bacterial strain closely related to *R. globerulus* species.

Despite the exceptional proprieties and the wide potential applications in different industrial sectors, the commercialization of biosurfactants remains costly and difficult. This is mainly due to the usage of expensive chemically synthesized media. Therefore, it is important to search for potential renewable, raw and cheap substrates to reduce the biosurfactant production cost [33]. The results here obtained showed that SP1d strain is able to grow and produce biosurfactants by using a wide range of different hydrophobic substrates such as exhausted vegetable oil and butter as sole source of carbon end energy.

The emulsification activity was often observed depending on the carbon source used for the bacterial cultivation [34]. Here, the results evidenced that n-hexadecane > diesel > exhausted vegetable oil > butter > motor oil were the best substrates in terms of production of crude biosurfactant. The biosurfactant production was confirmed by the value of surface tension reduction in the cell-free medium supplied with n-hexadecane, diesel and exhausted vegetable oil. Several manuscripts reported the production of surface-active compounds by both marine and terrestrial strains belonging to *Rhodococcus* genus using hydrocarbons as carbon sources [29, 35]. However, less information has been found regarding the other hydrophobic carbon sources. The production of biosurfactant on residual fried oil was already observed only in two *Rhodococcus erythropolis* strains reaching an E24 of about 60% and 40% by using 2–3% and 1% of substrate respectively [17, 36]. Different species of *Pseudomonas* and the marine *Nocardiopsis* sp. B4 were found to produce surface-active compounds from olive and sunflower oil [37–39]. Although the capability of a strain of *Rhodococcus* species of growing on butter and other animal fats has been already observed by Kis et al. [40], to the best of the authors’ knowledge, the production of surface-active compounds from these substrates by this genus have been not reported yet. Zouari et at [41]. described the synthesis of biosurfactants using buttermilk and poultry-transforming wastes by *Bacillus subtilis* SPB1, and active-surface compounds were produced with the animal fat waste by *Pseudomonas*, *Nocardia*, and *Aneurinibacillus* genera [42, 43]. Although used lubricating oils have become a serious environmental concern, only a few investigations were carried out to test these compounds as substrates for biosurfactant production. Mercade et al. [44] showed that among all the isolated strains only 10% of them synthesized biosurfactant utilizing waste lube oil. The phylogenetic characterization showed that all the strains belonged to *Rhodococcus* and *Bacillus* species. Moreover, the bacterial species *Corynebacterium kutscheri, Bacillus megaterium, Pseudomonas aeruginosa*, and the marine *Azotobacter chroococcum* showed the capability to grow and produce biosurfactants using waste motor oil lubricant as substrate [45–48].

Indeed, the results so far achieved evidenced that the biosurfactant produced by *Rhodococcus* sp. SP1d was able to emulsify a wide range of compounds such as diesel, olive oil, xylene, and n-hexadecane. The literature suggested that the emulsified activity of some *Rhodococcus* strains is mostly toward long-chain hydrocarbons [6]. The emulsification activity of SP1d was comparable or even higher in comparison with the other *Rhodococcus* strains [11, 17, 36].

Kinetics of biosurfactant production by strains belonging to *Rhodococcus* genus grown with hydrocarbons was widely studied, confirming the results here obtained. Under non-limiting conditions glycolipid production by *R. erythropolis* and *R. ruber* correlated with biomass formation and substrates consumption, suggesting its growth-associated mode [49, 50].

The results so far achieved evidenced that *Rhodococcus* sp. SP1d produced the highest amount of biosurfactant by using diesel and n-hexadecane as substrate. This observation suggests the potential use of this strain for bioremediation use. The surfactant production of *R. ruber* IEGM 231 and *R. wratislaviensis* BN38 increased with the increase in n-alkane chain length, reaching the maximal level of 9.9 and 3.1 g L^− 1^ respectively on n-hexadecane [50, 51]. Moreover, a yield of about 0.25–0.30 g L^− 1^ was reported in *R. erythropolis* S67 using n-hexadecane as a carbon end energy source [52]. Among four different carbon sources – crude oil, diesel, benzene, and naphthalene – the maximal biosurfactant production of *R. erythropolis* LMG 5359 and *R. ruber* LMG 5366 strains was obtained using naphthalene and diesel with yields of about 4.5–6 g L^− 1^. Although the growth of SP1d on diesel and n-hexadecane produced the highest amount of biosurfactant, this compound was even synthetized by using low-cost substrates such as exhausted vegetable oil (1.61 ± 0.23 g L^− 1^) and butter (0.81 ± 0.29 g L^− 1^). Synthesis of biosurfactants has been already investigated from waste frying oil. For instance, different studies reported a final production ranging from 0.8 to 2.7 g L^− 1^ [37, 38]. Moreover, it is reported that different factors such as C/N ratio and the use of different nitrogen sources can drastically affects biosurfactant synthesis [37, 38]. On the other hand, the culture conditions applied in this study were not optimized for biosurfactant biosynthesis. Further investigations should be carried out to maximize the production yields.

To investigate the potential use of biosurfactant synthetized by SP1d strain for environmental applications, its effect on the biofilm production of *P. protegens* MP12 was first evaluated. This latter strain exhibited PGP (Plant Growth Promotion) traits and inhibitory effects on mycelial growth towards grapevine phytopathogens such as *Aspergillus niger*, *Penicillium expansum, Neofusicoccum parvum*, *Botrytis cinerea*, and *Alternaria alternata* [23]. Leaf spraying of specific bacteria strains is a promising biological alternative to treatment with chemical pesticides against plant fungal diseases [53]. Previous observations demonstrated that *P. protegens* MP12 can be an appropriate biocontrol agent in epiphytic treatments [54]. The effectiveness of such protocol may be largely affected by the bacterial surface attachment through an high production of a persistent biofilm on the hydrophobic leaves surface. Biosurfactants can affect biofilm production in the freshwater microbial community [5]. For instance, rhamnolipid biosurfactants can reduce or increase biofilm production based on the belonging microbial species. Trehalose biosurfactant from *Rhodococcus fascians* BD8 showed antibiofilm activities against pathogenic bacteria such as *Escherichia coli*, *Vibrio harveyi*, and *Proteus vulgaris*, and yeast as *Candida albicans* [55]. As far as we know, the study here reported represents the first investigation on the effect of the trehalose biosurfactant in the biofilm production towards PGP bacteria such as *P. protegens* MP12. Here, it was found that 25 µg ml^− 1^ of biosurfactant from *Rhodococcus* sp. SP1d increased by a factor of ten the biofilm production in this strain. Our results encourage the use of this biosurfactant to improve adherence to plant surfaces and hence it may enhance the antifungal activity of *P. protegens* MP12.

Therefore, for a potential bioremediation application, the biosurfactant properties and chemical characterization were investigated using diesel as sole source of carbon and energy. In fact, although previous works showed the biosurfactant production by *Rhodococcus* genus using diesel as carbon source [29, 49] and its potential application in bioremediation protocols [22, 56], a detailed investigation of biosurfactants produced by this genus using diesel, one of the most environmental contaminants [57–59], as a sole source of carbon and energy is lacking in scientific literature.

The capability of *Rhodococcus* sp. SP1d to synthesize biosurfactants at both 10 and 18 °C is a great advantage when in-situ bioremediation protocols are applied in places where the temperature cannot be accurately controlled as in laboratory conditions. As already observed by Dang et al. [29] the results here achieved evidenced that biosurfactants production by *Rhodococcus* genus was not necessarily associated with bacterial growth on hydrocarbons but can be synthetized by resting cells. Here, the biosurfactant activity was maintained over wide NaCl concentrations, pH ranges, and heat treatments. However, a slight decrease of emulsifying activity was observed when purified biosurfactant was tested compared to cell-free medium. This aspect should be investigated in future studies. The stability of surface-active compounds synthesized by *Rhodococcus* genus to these stress conditions has been already documented [6, 11]. The effectiveness of biosurfactants under intense conditions of pH, NaCl, and temperature is a fundamental aspect to evaluate for their utilization in some industrial applications such as oil recovery and the bioremediation of polluted environment [60]. In fact, the literature reported that the biosurfactant exhibited superior properties if compared to the synthetic surfactants, that, for instance, are inactivated at 2–3% of salt [6]. Here, the results indicated that the crude biosurfactant purified from SP1d grown on diesel was able to form stable emulsions with both xylene and olive oil reaching an EA24 of about 55% by using 1000 mg L^− 1^ of crude biosurfactant. A purified trehalolipid (250 mg L^− 1^) synthesized by the marine bacterium *Rhodococcus* sp. PML026 was able to produce emulsions ranging from 34 to 46% that were stable in a wide range of temperatures and NaCl concentrations [22]. The literature evidenced an EA24 of about 50% and 60% with olive and crude oil respectively with 1000 mg L^− 1^ rhamnolipids produced by *P. aeruginosa* SP4 [61]. Eventually, the 250 mg L^− 1^ of surfactin produced by *Bacillus subtilis* showed an emulsification index of about 65% with diesel and kerosene and 55% with gasoline, suggesting higher stability of the emulsion formed with long-chained hydrocarbons. In fact, the emulsion formed with toluene presented a lower stability, since the EA24 value significantly decreased with time [62]. Here, a reduction of 25.0 mN m^− 1^ of surface tension was observed in water supplied with 1000 mg L^− 1^ of purified biosurfactant. This value is in accordance or slightly lower with measurements already reported in literature [17, 22, 29].

Analyses by NMR of the extracted biosurfactant were consistent with the compound being a trehalolipid comparable with other biosurfactants already isolated from *Rhodococcus* genus [22, 52]. In fact, the data obtained from the intact and hydrolyzed biosurfactant evidenced that the sugar moiety was trehalose linked with long-chain fatty acids saturated, unsaturated, and hydroxylated. Trehalolipids may find application in biomedical fields thanks to their anticancer, immunomodulation, antimicrobial, and antiviral properties [13]. Moreover, they are widely applied in environmental fields improving the solubility of hydrophobic compounds and desorption from contaminated soil [13, 63].

## Conclusions

This study reports the characterization of a new marine strain *Rhodococcus* sp. SP1d able to produce trehalolipid biosurfactant by using low-cost substrates such as butter and exhausted vegetable oils for reducing the costs of production. Moreover, results here obtained on diesel and n-hexadecane strongly suggest the potential application of this strain in bioremediation protocols in oil-polluted soil. Eventually, purified biosurfactant improved the biofilm formation of the PGPB *P. protegens* MP12, with potential application for increasing the adherence and colonization of plant surfaces and, thus, its antifungal activity.

## Materials and methods

### Cultivation of Rhodococcus sp. SP1d

*Rhodococcus* sp. SP1d was isolated from an aged hydrocarbon-polluted dredged sediment and screened for biosurfactant production as further described. The strain was routinely cultivated on Nutrient (Oxoid, Basingstoke, UK) broth (Nutrient 13 g L^-1^) or agar: nutrient broth supplemented with agar 15 g L^-1^ (Oxoid, Basingstoke, UK) at 27 °C (200 rpm).

### 16 S rDNA gene amplification and taxonomic analysis

The preliminar taxomonic identification of SP1d strain was carried out as described in Andreolli et al. [64]. Briefly, The DNA was purified from SP1d strain grown in 5 ml of Nutrient broth fro 48 h (27 °C at 200 rpm) by using Wizard^®^ Genomic DNA Purification Kit (Promega, Madison, WI, USA). The 16 S rDNA gene was amplified through fD1 and rP2 primers [65]. The PCR reaction was performed in a final volume of 25 µl containing ~ 100 ng of total DNA, 5× PCR buffer, 1 U of GoTaq^™^ DNA polymerase (Promega, Madison, WI, USA), 0.4 mM of dNTPs and 0.8 M of each primer. The amplification was carried out as follow: 95^°^C for 5 min, thus 30 cycles of 95^°^C for 1 min, 50 °C for 1 min, and 72 °C for 2 min, and a final extension at 72 °C for 5 min.

The 16S rRNA amplicon (1359 bp) was cloned in *Escherichia coli* Xl1-blue by using the Promega pGEM-T vector system (Promega, Madison, WI, USA) following the manual instructions. The gene was sequenced in both 5’ and 3’ direction by GATC Biotech (Cologne, Germany) and the nucleotide sequence was deposited in the NCBI database with the accession number MZ798796. The bacterial similarity was further searched through EzBioCloud database [66]. Therefore, the sequence alignment and the further phylogenetic tree based on the neighbor-joining algorithm were performed through MEGA-X software [67]. *Rhodococcus kroppenstedtii* K07-23^T^ was used as an out-group and bootstrap analysis was carried out on the basis of 1000 bootstrap replications.

### Growth curve with hydrophobic compounds

A starter culture of *Rhodococcus* sp. SP1d grown in Nutrient broth for 48 h was centrifuged (4,500 g for 15 min at 4 °C) and washed twice in physiological solution (0.9% NaCl in distilled water). Therefore, the cell suspension was transferred in 25 ml of a minimal defined medium (DM: Na_2_HPO_4_ 2.2 g L^− 1^, KH_2_PO_4_ 0.8 g L^− 1^, NH_4_NO_3_ 3 g L^− 1^) supplied with trace mineral and vitamin solutions as described in Frassinetti et al. [68] reaching a final OD_600_ of 0.01. The medium was supplied with 1% (*v/v*) of n-hexadecane, diesel (V-Power Diesel, Shell) exhausted sunflower oil (post-fried oil), or motor oil (Roil Petroli, Multigrade turbo SAE 10 W/40), previously sterilized by filtration. Moreover, butter sterilized by autoclave was used always reaching a final concentration of 1% (*w/v*). In the negative control samples, the substrate was omitted. The cultures were thus incubated at 27 °C for 10 days at 200 rpm. The growth of SP1d using diesel as substrate was monitored even at 18 and 10 °C. The cell counting was performed by plating serial dilution and the cell concentration was expressed by Colony Forming Unit (CFUs ml^− 1^). Data showed derived from three independent experiments.

### Investigation of the biosurfactants production on different growth substrates

The analysis of the biosurfactant production was carried out after 10 days of SP1d growth at 27 °C at 200 rpm in DM medium using n-hexadecane, diesel, sunflower exhausted oil, motor oil, and butter as sole sources of carbon and energy as above-described. Therefore, the culture was centrifuged at 10’000 g x for 10 min and the cell-free supernatant was used for the following tests.

### Oil-displacement assay

Oil-displacement test was carried out as previously described by Morikawa et al. [69]. Twenty ml of distilled water was added to a Petri dish, and crude petroleum oil was added until a thin film on the water surface was established. Then, 10 µl of cell-free culture was pipetted on the center of the oil film. Therefore, the diameter of the oil-free clearing zone was measured. Distilled water was used as the negative control. The assay was performed in triplicate.

### Emulsification activity (% EA24) and measurement of turbidity of the emulsion

The emulsification activity was assayed using diesel, olive oil, hexane, and xylene. Four ml of cell-free culture and 6 ml of test compounds were mixed for 3 min and, thus, it had been standing for 24 h at room temperature prior to measurement. The % EA24 was expressed as a percentage of the height of the emulsion layer divided by the total height of the mixture. In the case of the cell-free culture test, if enough emulsion was established (> 30% EA24) this phase was transferred using a Pasteur pipette to cuvettes, and absorbance was measured at 600 nm (Biophotometer, Eppendorf).

Additionally, the % EA24 was measured by adding 0, 250, 500, and 1000 mg L^− 1^ of biosurfactant purified from SP1d strain grown on diesel as the sole source of carbon and energy after 10 days of incubation to 2 ml of distilled water and 2 ml of olive oil or xylene.

### Surface tension measurement

A 10 ml of cell-free supernatant was put in a plate, placed onto the tensiometer platform and the surface tension was measured on a DY-300 instrument (Kyowa Interface Science Co, Niiza, Japan). The surface tension of the culture medium without bacterial inoculum was taken as reference. Additionally, the surface tension was determined in bidistilled water supplied with 1000 mg L^− 1^ of purified biosurfactant. Three readings for each sample were taken.

### Effect of biosurfactants on biofilm production of Pseudomonnas protegens MP12

The effect of biosurfactants on the biofilm production of *Pseudomonas protegens* MP12 [23] was investigated by using Calgary Biofilm Device (Innovotech, Edmonton Canada). It constitutes a 96-well microtiter plate and a lid with pegs protruding into the microtiter plate wells. The device was used to investigate the ability of the biosurfactant produced by *Rhodococcus* sp. SP1d as biofilm stimulating in the agro-industrial relevant strain *Pseudomonas protegens* MP12. Four different concentrations of biosurfactant produced by *Rhodococcus* sp. SP1d after 10 days of cultivation in DM supplied with 1% of n-hexadecane were tested. For the analysis, the microtiter plate was inoculated with *Pseudomonas protegens* MP12 at 0.05 and 1 OD_600_ in nutrient broth medium and double distilled water (i.e., ddH_2_O) respectively, and twofold serial dilutions between 200 and 25 mg ml^− 1^ of biosurfactant; bacteria growing with only nutrient broth (i.e., NB) and ddH_2_O were used as control. The microtiter was then incubated for 48 h at 27 °C and 150 rpm allowing biofilm production in both growing and resting cells. Experiments were performed in triplicate. To collect the biofilm produced, the peg lids were rinsed twice with physiological solution (i.e., 0.9% NaCl) to remove loosely bound cells and then placed into a new 96‐well plate containing 200 µl of physiological solution (i.e., recovery plate) in each well. Finally, it was sonicated for 30 min to remove biofilm from the peg to the recovery plate. Biofilm production was evaluated by tenfold serial dilution into physiological solution. The growth of planktonic cells not involved in biofilm production was measured before quantification of biofilm formation in 96-well plates. Viable cell numbers were determined using spot plate count and expressed as log_10_ colony forming units CFU mL^− 1^.

### Analysis of the biosurfactant production

The production of biosurfactants was monitored after 0, 48, 120, 168, and 240 h of incubation of *Rhodococcus* sp. SP1d at 27 °C (200 rpm) by using n-hexadecane, diesel, sunflower exhausted oil, motor oil, and butter as sole source of carbon and energy. Biosurfactant production was assessed by % EA24 as above-described using xylene and olive oil as reference compounds. The analysis was performed in triplicate.

#### Investigation of biosurfactant stability

The effect of heat, pH, and NaCl on the activity of the biosurfactant obtained both from crude extracted biosurfactant dissolved in water (1000 mg L^− 1^) and cell-free supernatant after 10 days of incubation (27 °C, 200 rpm) of *Rhodococcus* sp. SP1d using diesel as the sole source of carbon and energy was established. Different concentrations of NaCl from 0 to 25% (complete saturation; w/v) was applied. Moreover, the initial pH of cell-free broth (pH 6.6) was reduced and increased within the range of 2–12. To establish the heat stability of the biosurfactants, they were placed in a thermoblock set at 100 °C for different timing (from 0 to 120 min). Afterward, the samples were treated by autoclave at 121 ◦C for 15 min. After each treatment, the activity of the biosurfactant was determined by % EA24 measurement. The experimentation was performed in triplicate.

### Biosurfactant extraction and alkaline hydrolysis

The biosurfactants produced by *Rhodococcus* sp. SP1d using diesel as substrate were extracted as described by Kuyukina et al. [70]. A flask containing diesel without bacterial inoculum was even extracted as a control. Briefly, cultures of SP1d were centrifuged at 10,000 g for 10 min at 4 °C, and the hydrophobic phase layer at the surface was transferred in a new flask. Biosurfactants were extracted by using methyl tertiary-butyl ether (MTBE) as a solvent system at room temperature on a rotary shaker (200 rpm) for 3 h. The solvent layer was separated from the aqueous phase through a separating funnel, and the solvent was removed by freeze-dried and stored at – 80 °C.

The alkaline hydrolysis was performed for 2 h in 1 M NaOH at 90 °C with regular mixing. After cooling, an equal volume of 1 M HCl was added. Therefore, a 1:1 volume of hexane was supplied, vigorously mixed end centrifuged. The upper hexane and the lower water layers were separated and lyophilized.

#### Nuclear magnetic resonance spectroscopy (NMR)

NMR experiments were acquired on a Bruker Advance III 600 spectrometer, operating at 600.13 MHz proton Larmor frequency, and equipped with a triple resonance TCI cryoprobe. All the spectra were recorded using Bruker-standard pulse sequences. Two-dimensional (2D) homonuclear ^1^ H-^1^ H total correlation spectroscopy (TOCSY) spectra were recorded with a mixing time of 60 ms, 8 transients, and a spectral window of 10 204 Hz in both dimensions, collecting 512 t_1_ increments, each consisting of 2048 complex points. The heteronuclear 2D ^1^ H-^13^ C Heteronuclear Single Quantum Coherence (HSQC) spectra were recorded with 16 transients, a spectral window of 12,019 Hz in the ^1^ H and 21,127 Hz in the ^13^ C dimensions, collecting 256 t_1_ increments, each consisting of 1024 complex points. The heteronuclear 2D ^1^ H-^13^ C Heteronuclear Multiple Bond Correlation (HMBC) spectra were recorded with 64 transients, a spectral window of 7211 Hz in the ^1^ H and 33,201 Hz in the ^13^ C dimensions, collecting 256 t_1_ increments, each consisting of 4096 complex points, and using J_CH_ long-range coupling of 8 Hz.

All spectra were manually phased and baseline-corrected using TOPSPIN 3.6.1 (Bruker) and were referenced to trimethylsilylpropanoic acic (TSP) signal at 0 ppm. Samples were dissolved in D_2_O (crude biosurfactants and aqueous phase after basic hydrolysis) or deuterated methanol (organic phase after basic hydrolysis). All the spectra were acquired at 25 °C.

#### Acknowledges

Centro Piattaforme Tecnologiche of the University of Verona is acknowledged for providing access to the NMR instrument.

## Data Availability

All data generated or analysed during this study are included in this published article.

## References

[1] Liu K, Sun Y, Cao M, Wang J, Lu JR, Xu H (2020). Rational design, properties and applications of biosurfactants: a short review of recent advances. Curr Opin Colloid Interface Sci.

[2] Mujumdar S, Joshi P, Karve N (2019). Production, characterization, and applications of bioemulsifiers (BE) and biosurfactants (BS) produced by *Acinetobacter* spp.: a review. J Basic Microbiol.

[3] Jahan R, Bodratti AM, Tsianou M, Alexandridis P. Biosurfactants, natural alternatives to synthetic surfactants: physicochemical properties and applications. Adv Colloid Interface Sci. 2019;102061. 10.1016/j.cis.2019.102061. 10.1016/j.cis.2019.102061 31767119

[4] Roy A (2017). A review on the biosurfactants: properties, types and its applications. J Fundam Renew Energ Appl.

[5] Gill SP, Hunter WR, Coulson LE, Banat IM, Schelker J (2022). Synthetic and biological surfactant effects on freshwater biofilm community composition and metabolic activity. Appl Microbiol Biotechnol.

[6] Shavandi M, Mohebali G, Haddadi A, Shakarami H, Nuhi A (2011). Emulsification potential of a newly isolated biosurfactant-producing bacterium, *Rhodococcus* sp. strain TA6. Colloids Surf B.

[7] Andreolli M, Lampis S, Brignoli P, Vallini G (2021). Mesocosm-based simulations to optimize a bioremediation strategy for the effective restoration of wildfire‐impacted soils contaminated with high‐molecular‐weight hydrocarbons. J Appl Microbiol.

[8] Banat IM, Franzetti A, Gandolfi I, Bestetti G, Martinotti MG, Fracchia L, Smyth TJ, Marchant R (2010). Microbial biosurfactants production, applications and future potential. Appl Microbiol Biotechnol.

[9] Bouchez-Naïtali M, Rakatozafy H, Marchal R, Leveau JY, Vandecasteele JP (1999). Diversity of bacterial strains degrading hexadecane in relation to the mode of substrate uptake. J Appl Microbiol.

[10] Iwabuchi N, Sunairi M, Anzai H, Nakajima M, Harayama S (2000). Relationships between colony morphotypes and oil tolerance in *Rhodococcus rhodochrous*. Appl Environ Microbiol.

[11] Kuyukina MS, Ivshina IB, Alvarez HM (2019). Production of trehalolipid biosurfactants by *Rhodococcus*. Biology of *Rhodococcus*.

[12] Liu CW, Liu HS (2011). *Rhodococcus erythropolis* strain NTU-1 efficiently degrades and traps diesel and crude oil in batch and fed-batch bioreactors. Process Biochem.

[13] Franzetti A, Gandolfi I, Bestetti G, Smyth TJ, Banat IM (2010). Production and applications of trehalose lipid biosurfactants. Eur J Lipid Sci Technol.

[14] Mnif I, Ellouz-Chaabouni S, Ghribi D (2018). Glycolipid biosurfactants, main classes, functional properties and related potential applications in environmental biotechnology. J Polym Environ.

[15] Ciapina EM, Melo WC, Santa Anna LM, Santos AS, Freire DM, Pereira N (2006). Biosurfactant production by *Rhodococcus erythropolis* grown on glycerol as sole carbon source. Appl Biochem Biotechnol.

[16] Ruggeri C, Franzetti A, Bestetti G, Caredda P, La Colla P, Pintus M, Sergi S, Tamburini E (2009). Isolation and characterisation of surface active compound-producing bacteria from hydrocarbon-contaminated environments. Int Biodeterior Biodegr.

[17] Sadouk Z, Hacene H, Tazerouti A (2008). Biosurfactants production from low cost substrate and degradation of diesel oil by a *Rhodococcus* strain. Oil Gas Sci Technol.

[18] Hassanshahian M, Ahmadinejad M, Tebyanian H, Kariminik A (2013). Isolation and characterization of alkane degrading bacteria from petroleum reservoir waste water in Iran (Kerman and Tehran provenances). Mar Pollut Bull.

[19] Hassanshahian M (2014). Isolation and characterization of biosurfactant producing bacteria from Persian Gulf (Bushehr provenance). Mar Pollut Bull.

[20] Maneerat S (2005). Biosurfactants from marine microorganisms. Songklanakarin J Sci Technol.

[21] Satpute SK, Banat IM, Dhakephalkar PK, Banpurkar AG, Chopade BA (2010). Biosurfactants, bioemulsif[iers and exopolysaccharides from marine microorganisms. Biotechnol Adv.

[22] White DA, Hird LC, Ali ST (2013). Production and characterization of a trehalolipid biosurfactant produced by the novel marine bacterium *Rhodococcus* sp., strain PML026. J Appl Microbiol.

[23] Andreolli M, Zapparoli G, Angelini E, Lucchetta G, Lampis S, Vallini G (2019). *Pseudomonas protegens* MP12: a plant growth-promoting endophytic bacterium with broad-spectrum antifungal activity against grapevine phytopathogens. Microbiol Res.

[24] Ulrich EL, Hideo A, Jurgen FD, Yoko H, Yannis EI, Jundong L, Miron L, Mading S, Maziuk D, Miller Z, Nakatani E, Schulte CF, Tolmie DE, Wenger RK, Yao H, Markley JL (2007). BioMagResBank Nucleic Acids Res.

[25] Bubb WA (2003). NMR spectroscopy in the study of carbohydrates: characterizing the structural complexity. Concepts Magn Reson Part A Educ J.

[26] Alexandri E, Ahmed R, Siddiqui H, Choudhary MI, Tsiafoulis CG, Gerothanassis IP (2017). High resolution NMR spectroscopy as a structural and analytical tool for unsaturated lipids in solution. Molecules.

[27] Tripathi L, Irorere VU, Marchant R, Banat IM (2018). Marine derived biosurfactants: a vast potential future resource. Biotechnol Lett.

[28] Thavasi R, Jayalakshmi S, Banat IM (2011). Effect of biosurfactant and fertilizer on biodegradation of crude oil by marine isolates of *Bacillus megaterium*, *Corynebacterium kutscheri* and *Pseudomonas aeruginosa*. Bioresour Technol.

[29] Dang NP, Landfald B, Willassen NP (2016). Biological surface-active compounds from marine bacteria. Environ Technol.

[30] Hentati D, Chebbi A, Loukil S, Kchaou S, Godon JJ, Sayadi S, Chamkha M (2016). Biodegradation of fluoranthene by a newly isolated strain of *Bacillus stratosphericus* from Mediterranean seawater of the Sfax fishing harbour, Tunisia. Environ Sci Pollut Res Int.

[31] Mapelli F, Scoma A, Michoud G, Aulenta F, Boon N, Borin S, Kalogerakis N, Daffonchio D (2017). Biotechnologies for marine oil spill cleanup: indissoluble ties with microorganisms. Trends Biotechnol.

[32] Ortega-de la Rosa ND, Vázquez-Vázquez JL, Huerta-Ochoa S, Gimeno M, Gutie´rrez-Rojas M (2018). Stable bioemulsifiers are produced by *Acinetobacter bouvetii* UAM25 growing in different carbon sources. Bioprocess Biosyst Eng.

[33] Banat IM, Satpute SK, Cameotra SS, Patil R, Nyayanit NV (2014). Cost effective technologies and renewable substrates for biosurfactants’ production. Front Microbiol.

[34] Malavenda R, Rizzo C, Michaud L, Gerçe B, Bruni V, Syldatk C, Hausmann R, Giudice AL (2015). Biosurfactant production by Arctic and Antarctic bacteria growing on hydrocarbons. Polar Biol.

[35] Pi Y, Chen B, Bao M, Fan F, Cai Q, Ze L, Zhang B (2017). Microbial degradation of four crude oil by biosurfactant producing strain *Rhodococcus* sp. Bioresour Technol.

[36] Pirog T, Sofilkanych A, Konon A, Shevchuk T, Ivanov S (2013). Intensification of surfactants’ synthesis by *Rhodococcus erythropolis* IMV Ac-5017, *Acinetobacter calcoaceticus* IMV B-7241 and *Nocardia vaccinii* K-8 on fried oil and glycerol containing medium. Food Bioprod Process.

[37] Abouseoud M, Maachi R, Amrane A (2007). Biosurfactant production from olive oil by *Pseudomonas fluorescens*. Comm Curr Res Educ Top Trends Appl Microbiol.

[38] Haba E, Espuny MJ, Busquets M, Manresa A (2000). Screening and production of rhamnolipids by *Pseudomonas aeruginosa* 47T2 NCIB40044 from waste frying oils. J Appl Microbiol.

[39] Khopade A, Biao R, Liu X, Mahadik K, Zhang L, Kokare C (2012). Production and stability studies of the biosurfactant isolated from marine *Nocardiopsis* sp. B4. Desalination.

[40] Kis Á, Laczi K, Zsíros S, Rákhely G, Perei K (2015). Biodegradation of animal fats and vegetable oils by *Rhodococcus erythropolis* PR4. Int Biodeterior Biodegr.

[41] Zouari R, Ellouze-Chaabouni S, Ghribi D (2019). Use of butter milk and poultry-transforming wastes for enhancing production of *Bacillus subtilis* SPB1 biosurfactant in submerged fermentation. J Microbiol Biotechnol Food Sci.

[42] Ramani KS, Jain SC, Mandal AB, Sekaran G (2012). Microbial induced lipoprotein biosurfactant from slaughterhouse lipid waste and its application to the removal of metal ions from aqueous solution. Colloids Surf B: Biointerfaces.

[43] Sellami M, Khlifi A, Frikha F, Miled N, Belbahri L, Rebah FB (2016). Agro-industrial waste based growth media optimization for biosurfactant production by *Aneurinibacillus migulanus*. J Microbiol Biotechnol Food Sci.

[44] Mercade ME, Monleon L, De Andres C, Rodon I, Martinez E, Espuny MJ, Manresa A (1996). Screening and selection of surfactant-producing bacteria from waste lubricating oil. J Appl Bacteriol.

[45] Thavasi R, Jayalakshmi S, Balasubramanian T, Banat IM (2007). Biosurfactant production by Corynebacterium kutscheri from waste motor lubricant oil and peanut oil cake. Lett Appl Microbiol.

[46] Thavasi R, Jayalakshmi S, Balasubramanian T, Banat IM (2008). Production and characterization of a glycolipid biosurfactant from *Bacillus megaterium* using economically cheaper sources. World J Microbiol Biotechnol.

[47] Thavasi R, Nambaru VS, Jayalakshmi S, Balasubramanian T, Banat IM (2009). Biosurfactant production by *Azotobacter chroococcum* isolated from the marine environment. Mar Biotechnol.

[48] Thavasi R, Nambaru VS, Jayalakshmi S, Balasubramanian T, Banat IM (2011). Biosurfactant production by *Pseudomonas aeruginosa* from renewable resources. Indian J Microbiol.

[49] Haddadin MS, Arqoub AAA, Reesh IA, Haddadin J (2009). Kinetics of hydrocarbon extraction from oil shale using biosurfactant producing bacteria. Energy Convers Manag.

[50] Philp JCMS, Kuyukina M, Ivshina I, Dunbar S, Christofi N, Lang S, Wray V (2002). Alkanotrophic *Rhodococcus ruber* as a biosurfactant producer. Appl Microbiol Biotechnol.

[51] Tuleva B, Christova N, Cohen R, Stoev G, Stoineva I (2008). Production and structural elucidation of trehalose tetraesters (biosurfactants) from a novel alkanothrophic *Rhodococcus wratislaviensis* strain. J Appl Microbiol.

[52] Luong TM, Ponamoreva ON, Nechaeva IA, Petrikov KV, Delegan YA, Surin AK, Linklater D, Filonov AE (2018). Characterization of biosurfactants produced by the oil-degrading bacterium *Rhodococcus erythropolis* S67 at low temperature. World J Microbiol Biotechnol.

[53] Ganeshan G, Manoj Kumar A (2005). *Pseudomonas fluorescens*, a potential bacterial antagonist to control plant diseases. J Plant Interact.

[54] Andreolli M, Zapparoli G, Lampis S, Santi C, Angelini E, Bertazzon N (2021). In vivo endophytic, rhizospheric and epiphytic colonization of *Vitis vinifera* by the plant-growth promoting and antifungal strain *Pseudomonas protegens* MP12. Microorganisms.

[55] Janek T, Krasowska A, Czyżnikowska Ż, Łukaszewicz M (2018). Trehalose lipid biosurfactant reduces adhesion of microbial pathogens to polystyrene and silicone surfaces: an experimental and computational approach. Front Microbiol.

[56] Ivshina I, Kostina L, Krivoruchko A, Kuyukina M, Peshkur T, Anderson P, Cunningham C (2016). Removal of polycyclic aromatic hydrocarbons in soil spiked with model mixtures of petroleum hydrocarbons and heterocycles using biosurfactants from *Rhodococcus ruber* IEGM 231. J Hazard Mater.

[57] Andreolli M, Lampis S, Brignoli P, Vallini G (2016). *Trichoderma longibrachiatum* Evx1 is a fungal biocatalyst suitable for the remediation of soils contaminated with diesel fuel and polycyclic aromatic hydrocarbons. Environ Sci Pollut Res.

[58] Doni S, Macci C, Martinelli C, Iannelli R, Brignoli P, Lampis S, Andreolli M, Vallini G, Masciandaro (2018). Combination of sediment washing and bioactivators as a potential strategy for dredged marine sediment recovery. Ecol Eng.

[59] Khalid FE, Lim ZS, Sabri S, Gomez-Fuentes C, Zulkharnain A, Ahmad SA (2021). Bioremediation of diesel contaminated marine water by bacteria: a review and bibliometric analysis. J Mar Sci Eng.

[60] Banat IM (1995). Biosurfactants production and possible uses in microbial enhanced oil recovery and oil pollution remediation: a review. Bioresour Technol.

[61] Pornsunthorntawee O, Wongpanit P, Chavadej S, Abe M, Rujiravanit R (2008). Structural and physicochemical characterization of crude biosurfactant produced by *Pseudomonas aeruginosa* SP4 isolated from petroleum-contaminated soil. Bioresour Technol.

[62] Felix AKN, Martins JJ, Almeida JGL, Giro MEA, Cavalcante KF, Melo VMM, Pessoa ODL, Rocha MVP, Gonçalves LRB, de Santiago Aguiar RS (2019). Purification and characterization of a biosurfactant produced by *Bacillus subtilis* in cashew apple juice and its application in the remediation of oil-contaminated soil. Colloids Surf B.

[63] Chang JS, Radosevich M, Jin Y, Cha DK (2004). Enhancement of phenanthrene solubilization and biodegradation by trehalose lipid biosurfactants. Environ Toxicol Chem Int J.

[64] Andreolli M, Lampis S, Bernardi P, Calò S, Vallini G (2020). Bacteria from black crusts on stone monuments can precipitate CaCO_3_ allowing the development of a new bio-consolidation protocol for ornamental stone. Int Biodeterior Biodegr.

[65] Weisburg WG, Barns SM, Pelletier DA, Lane DJ (1991). 16S ribosomal DNA amplification for phylogenetic study. J Bacteriol.

[66] Seok-Hwan Y, Sung-Min H, Soonjae K, Jeongmin L, Yeseul K, Hyungseok S, Jongsik C (2017). Introducing EzBioCloud: a taxonomically united database of 16S rRNA gene sequences and whole-genome assemblies. Int J Syst Evol Microbiol.

[67] Kumar S, Stecher G, Li M, Knyaz C, Tamura K (2018). MEGA X: molecular evolutionary genetics analysis across computing platforms. Mol Biol Evol.

[68] Frassinetti S, Setti L, Corti A, Farinelli P, Montevecchi P, Vallini G (1998). Biodegradation of dibenzothiophene by a nodulating isolate of *Rhizobium meliloti*. Can J Microbiol.

[69] Morikawa M, Hirata Y, Imanaka T (2000). A study on the structure–function relationship of lipopeptide biosurfactants. Biochim Biophys Acta Mol Cell Biol Lipids.

[70] Kuyukina MS, Ivshina IB, Philp JC, Christofi N, Dunbar SA, Ritchkova MI (2001). Recovery of *Rhodococcus* biosurfactants using methyl tertiary-butyl ether extraction. J Microbiol Methods.

